# Anticancer Drug Discovery from Microbial Sources: The Unique Mangrove Streptomycetes

**DOI:** 10.3390/molecules25225365

**Published:** 2020-11-17

**Authors:** Jodi Woan-Fei Law, Lydia Ngiik-Shiew Law, Vengadesh Letchumanan, Loh Teng-Hern Tan, Sunny Hei Wong, Kok-Gan Chan, Nurul-Syakima Ab Mutalib, Learn-Han Lee

**Affiliations:** 1 Novel Bacteria and Drug Discovery (NBDD) Research Group, Microbiome and Bioresource Research Strength, Jeffrey Cheah School of Medicine and Health Sciences, Monash University Malaysia, Bandar Sunway 47500, Selangor Darul Ehsan, Malaysia; jodi.law1@monash.edu (J.W.-F.L.); Vengadesh.Letchumanan1@monash.edu (V.L.); loh.teng.hern@monash.edu (L.T.-H.T.); 2 Monash Credentialed Pharmacy Clinical Educator, Faculty of Pharmacy and Pharmaceutical Sciences, Monash University, 381 Royal Parade, Parkville 3052, VIC, Australia; lydia19595@gmail.com; 3 Li Ka Shing Institute of Health Sciences, Department of Medicine and Therapeutics, The Chinese University of Hong Kong, Shatin, Hong Kong, China; wonghei@cuhk.edu.hk; 4 Division of Genetics and Molecular Biology, Institute of Biological Sciences, Faculty of Science, University of Malaya, Kuala Lumpur 50603, Malaysia; 5 International Genome Centre, Jiangsu University, Zhenjiang 212013, China; 6 UKM Medical Molecular Biology Institute (UMBI), UKM Medical Centre, Universiti Kebangsaan Malaysia, Kuala Lumpur 56000, Malaysia

**Keywords:** mangrove, anticancer, cytotoxic, bioactive, *Streptomyces*

## Abstract

Worldwide cancer incidence and mortality have always been a concern to the community. The cancer mortality rate has generally declined over the years; however, there is still an increased mortality rate in poorer countries that receives considerable attention from healthcare professionals. This suggested the importance of the prompt detection, effective treatment, and prevention strategies. The genus *Streptomyces* has been documented as a prolific producer of biologically active secondary metabolites. Streptomycetes from mangrove environments attract researchers’ attention due to their ability to synthesize diverse, interesting bioactive metabolites. The present review highlights research on mangrove-derived streptomycetes and the production of anticancer-related compounds from these microorganisms. Research studies conducted between 2008 and 2019, specifically mentioning the isolation of streptomycetes from mangrove areas and described the successful purification of compound(s) or generation of crude extracts with cytotoxic activity against human cancer cell lines, were compiled in this review. It is anticipated that there will be an increase in prospects for mangrove-derived streptomycetes as one of the natural resources for the isolation of chemotherapeutic agents.

## 1. Introduction

In this rapidly changing world, accompanied by an urbanized lifestyle and alteration in dietary habits, our health has inevitably impacted in both positive and negative ways. Unfortunately, cancer remains a global health issue, and it has been recorded as the second leading cause of death following cardiovascular disease [1,2]. There are many speculative causes of cancer that are often related to contemporary unhealthy lifestyles, such as dietary changes towards the consumption of fast food and alcohol, smoking, and the lack of physical activity [3,4,5,6]. The documented global cancer cases were 24.5 million, and the estimated cancer death was 9.6 million in the year 2017 [7]. The continuous burden of cancer has been depicted in the report written by Siegel et al. [8]. As of the year 2020, the most common estimated new cases of cancers for Americans based on sex: (a) for men will be prostate, lung, and bronchus, and colorectal cancers; (b) while for women will be breast, lung, and colorectal cancers. Cancer deaths would be at an alarming rate of approximately 1600 deaths per day [8].

Human cancer is a complex process involving cellular and molecular alterations mediated by numerous endogenous and exogenous stimuli [9,10,11,12]. In other words, these stimuli cause the uncontrollable multiplication of cells that become cancerous once they no longer obey normal cell signalling and start to invade other tissues [13,14,15]. Several factors contribute to the progress in reducing cancer-related mortality, such as early detection, effective treatment through chemotherapy, radiation therapy or surgical procedure, and prevention [16,17,18,19]. Among these, the search for effective anticancer drugs is a paramount goal for cancer therapy.

Natural products from various sources, including plants, environments, and microorganisms, have been serving us well in combating cancer. Approximately more than 60% of the present anticancer/antitumor drugs were naturally derived from these sources [20,21,22,23,24]. In general, the phrase “natural product” can be referred to as “primary or secondary metabolites,” biologically active compounds with small molecular weight (less than 3000 Daltons) that are produced by organisms to assist in their survival [25,26,27]. Natural products have the potential to suppress or inhibit cancer progression as well as involve the reversal of progression [16,28]. Natural products could also be an alternative solution to overcome current chemotherapy issues such as multidrug resistance, distressing side effects (e.g., heart failure, diarrhoea, oedema, etc.) due to high toxicity or lack of specificity [29,30,31,32]. Ideally, chemotherapeutic medicines should be highly specific in eliminating only cancer cells by discriminating between the normal cells and cancer cells. However, most of the presently used anticancer treatments tend to destroy cancer and normal cells [33]. Cancer chemoprevention is equally important as an intervention in carcinogenesis. These can be blocking agents that halt neoplastic process or suppressing agents that prevent the development of cancer cells’ malignant phenotype [34,35]. Thus, it is an ongoing effort to search for highly specific and potent chemotherapy/chemopreventive agents from alternative sources such as microorganisms.

## 2. Microbial Sources: Why the *Streptomyces*?

Microorganisms have been established as essential resources for natural product drug discovery. The abundance of species can be the root of the enormous supply of structurally diverse natural products. By harvesting these compounds from microbial sources, it will be relatively cost-efficient compared to the chemical synthesis approach [36,37,38]. As a result, many microorganisms have been screened for the production of anticancer compounds/leads. Purportedly, these microbial natural compounds’ anticancer activity could regulate immune function, inhibit cell proliferation, and induce apoptosis [39,40].

In the *Bacteria* domain, the phylum *Actinobacteria* is one of the renowned major lineages representing one of the largest taxonomic units with diverse identified species and some of which are well studied model organisms [41,42,43]. This phylum consists of six major classes: *Acidimicrobiia*, *Actinobacteria*, *Coriobacteriia*, *Nitriliruptoria*, *Rubrobacteria*, and *Thermoleophilia*; covering a wide range of Gram-positive bacteria with different characteristics and morphologies [44,45,46]. *Actinobacteria* have been recognized as primary sources of bioactive natural products as early as in the 1950s, for which approximately half of the secondary metabolites discovered, including enzymes, antibiotics, immunosuppressive, and anti-tumour agents, are produced by actinomycetes [41,47,48,49,50,51]. The well known representative genus of class *Actinobacteria* is the *Streptomyces*, which accounts for over 70% of commercially useful antibiotics [48,52,53]. In addition, it is remarkable that 80% of actinobacterial natural products documented hitherto are derived from the genus *Streptomyces* [47].

Streptomycetes have immense diversity and bioactive potential that overpower many other microbial groups. For instance, streptomycetes are extensively distributed in both terrestrial and marine habitats, which to date, this group of bacteria has more than 900 species identified with a validly published name (https://bacterio.net/). Additionally, an increasing number of studies indicate the existence of streptomycetes in extreme habitats [43,54,55,56]. Streptomycetes produce a high number of spores that are easily dispersed, and thus, it is projected that they have a cosmopolitan distribution [47,57]. Furthermore, the biosynthetic capacity of streptomycetes remains without rival given the fact that streptomycetes have a large genome size (>7 Mbp) [58,59,60,61], and some of them have been previously reported to own more than 20 gene clusters encoding predicted/known biosynthetic pathways [62,63,64]. Hence, this contributed to the unique capability of streptomycetes in the production of various novel and/or useful secondary metabolites with antioxidant, anticancer, antimicrobial, etc. activities which subsequently become the key reason which attracts researchers to explore the bioactive potential of this genus [65,66,67]. Although only limited numbers of the isolated compounds from microbes can be directly utilized as clinically effective medicines in their own right (naturally occurring form), streptomycetes are verified producers of anticancer drugs such as bleomycin (glycopeptides group), dactinomycin (non-ribosomal peptides group), mitomycin C (quinones group), and doxorubicin (anthracyclines group) [68,69]. Furthermore, natural microbial compounds can be optimized/modified and developed into important drug leads [70].

## 3. Bioprospecting of *Streptomyces* spp. from Mangrove Environment

Several research studies have adapted the culture-dependent bioprospecting strategy where the microorganisms are initially isolated from neglected or extreme habitats in the journey of unearthing novel natural products from streptomycetes as potential pharmacological agents, such as from mangroves, deserts, and caves [66,71,72,73,74,75]. Generally, this strategy involves utilizing appropriate selective isolation procedures to obtain microorganisms of interest from a neglected habitat, followed by the recognition and dereplication of target microbes and then the selection of representative strains for bioactivity screening purposes. Nonetheless, it should be noted that this strategy relies on the concept that poorly studied habitats can be potential sources for the isolation of novel strains that could produce new and interesting compounds [71,76].

Apart from terrestrial and aquatic environments, streptomycetes’ distribution in underexplored dynamic mangrove environments appears to be relatively extensive, and bioprospecting studies of streptomycetes from mangrove areas are generating fruitful outcomes [47,66,77]. Mangroves are special intertidal ecosystems of tropical and subtropical regions [78,79,80]. Mangrove forests act as highly productive ecosystems providing crucial protections to the coastline in tidal zones, homes to diverse species of marine, freshwater, and terrestrial flora and fauna, as well as ecosystem goods to sustain human wellbeing [48,81,82]. Moreover, this environment contains a rich amount of nutrients due to nutrient cycling between the microbial community and mangrove vegetation; thus, this cycle further promotes the mangrove as a homeland to various microorganisms [83].

There is increasing evidence regarding the utilization of mangrove microorganism resources, which could be due to these microorganisms’ ability to produce a vast array of unusual secondary metabolites driven by inconsistent environmental factors such as a salinity and tidal gradient that is believed to prompt metabolic pathway adaptations in them [48,84,85]. Streptomycetes are successful producers of over 10,000 bioactive compounds. The bioactive potential of mangrove-derived streptomycetes has been of scientific interest since the mangrove ecosystem has become a hot spot for natural product discovery [47,86,87,88]. Research has also proven that mangroves serve as invaluable assets by offering diversified novel taxa. Numerous new species have been first discovered from mangroves and described by researchers around the world. For example, *Streptomyces nigra* and *Streptomyces avicenniae* were isolated from mangrove *Avicennia marina* in Fujian Province, China, and characterized as a novel species by Chen et al. [89] and Xiao et al. [90] respectively; *Streptomyces sundarbansensis* was first discovered as a novel species from the sediments of mangrove Sundarbans, India, by Arumugam et al. [91]; *Streptomyces xiamenensis* was described as novel species by Xu et al. [92] and it was isolated from a mangrove forest in Fujian Province, China. Screenings on the bioactivity of secondary metabolites from mangrove-derived streptomycetes have been conducted. The findings revealed that bioactivities such as antibacterial, antifungal, anti-HIV, anticancer, and antioxidant had been demonstrated by these isolates [77,93,94,95,96,97].

## 4. Discovery of Novel Compounds with Anticancer/Cytotoxic Activity from Mangrove-Derived *Streptomyces* spp.

The screening for anticancer potentials of mangrove-derived streptomycetes has been an ongoing expedition for over 10 years. The present review compiled studies carried out specifically on streptomycetes from mangrove areas that demonstrated cytotoxic activity against human cancer cell lines (Table 1). These studies consisted of experiments conducted on pure compounds or crude extracts of diverse *Streptomyces* spp. which have been isolated from mangrove environments. All studies summarized in Table 1 had utilized the 3-(4,5-dimethylthiazol-2-yl)-2,5 diphenyl tetrazolium bromide (MTT) assay to analyse the cytotoxic capacity of a compound or extract against different human cancer cells. The MTT assay is based on the concept of living cells capable of converting MTT into formazan crystals, hence, reflecting the activity of mitochondria in living cells. For most cell populations, the total activity of mitochondria is linearly associated with the number of viable cells. The formazan crystals can be solubilized for homogenous measurement whereby formazan concentration reflected in optical density determines the number of viable cells. The MTT assay is the most commonly used method in determining drug cytotoxicity at different concentrations. The advantage of the MTT assay is that it serves to measure viable cells in a high-throughput manner (96-well plates) without requiring the complex counting of cells [98].

According to the information collected in Table 1, many studies provided compelling evidence that these streptomycetes are good sources for isolating anticancer-related compounds. One example of an earlier study was conducted by Xie et al. [99], where they successfully purified and elucidated five compounds derived from a mangrove *Streptomyces* strain 124092. Among these compounds, four of them exhibited cytotoxic activity against the human hepatoma SMMC-7721 cell line (Table 1). Additionally, new compounds with anticancer potential have been discovered from mangrove-derived streptomycetes. For instance, Yuan et al. [100] isolated two novel compounds from mangrove *Streptomyces* sp. 211726: azalomycin F_4a_ 2-ethylpentyl ester and azalomycin F_5a_ 2-ethylpentyl ester, where both compounds exhibited strong cytotoxic activity against the HCT-116 colon cancer cell line with IC_50_ values as low as 5.64 µg/mL and 2.58 µg/mL, respectively. Furthermore, Fu et al. [101] discovered streptocarbazoles A and B—novel indolocarbazoles isolated from *Streptomyces* sp. FMA. Streptocarbazole A exhibited impressive cytotoxic effect against human cancer cells including leukaemia HL-60 cells (IC_50_ = 1.4 µM or 0.67 µg/mL), lung cancer A549 cells (IC_50_ = 5.0 µM or 2.41 µg/mL), and HeLa cells (IC_50_ = 34.5µM or 16.61 µg/mL), while the streptocarbazole B exhibited a cytotoxic effect against HeLa cells (IC_50_ = 22.5µM or 10.47 µg/mL) only. Further analysis revealed that streptocarbazole A (10 µM) caused the cell cycle arrest of HeLa cells in the G_2_/M phase [101]. Recently, halichoblelide D—a new elaiophylin derivative—was one of the bioactive secondary metabolites isolated from a mangrove strain *Streptomyces* sp. 219807. It was found that this new compound exerted significant cytotoxicity against HeLa cells (IC_50_ = 0.3 µM or 0.27 µg/mL and MCF-7 cells (IC_50_ = 0.33 µM or 0.30 µg/mL) among other known strong cytotoxic compounds that were also isolated, such as 2-methylelaiophylin (IC_50_ = 0.29 µM or 0.30 µg/mL against both HeLa and MCF-7 cells) and elaiophylin (IC_50_ = 0.29 µM or 0.30 µg/mL against HeLa cells; IC_50_ = 0.19 µM or 0.19 µg/mL against MCF-7 cells) [102].

Nevertheless, some studies utilized assays aside from MTT for the evaluation of the cytotoxic activity of the compounds, for examples, the sulforhodamine B (SRB) method, cell counting kit-8 (CCK-8) (Dojindo) assay, and the lactate dehydrogenase (LDH) assay. The SRB method estimates the total cellular protein content, which is based on the ability of SRB dye binding in a pH-dependent and electrostatic manner on protein basic amino acid residues of trichloroacetic acid (TCA)-fixed cells in mild acidic conditions. Weak bases including Tris base enable SRB extraction quantitatively from cells and solubilize SRB for optical density measurement. Therefore, the number of viable cells and cellular protein measured are linearly related [103,104]. For instance, a study conducted by Chen et al. [105] utilized the SRB assay to evaluate the cytotoxic activity of several novel compounds isolated from a mangrove streptomycete. Novel bagremycins C–E, phenol esters of p-hydroxystyrene and p-hydroxybenzoic acid, were produced by *Streptomyces* sp. Q22, where bagremycin C was active against human glioma cell lines U87MG (IC_50_ = 2.2 µM), U251 (IC_50_ = 4.3 µM), and SHG44 (IC_50_ = 2.4 µM) [105].

The CCK-8 assay is a colorimetric assay utilized to measure the in vitro cell viability for cell cytotoxicity and proliferation experimentation. The assay operates upon the reduction of Dojindo’s water-soluble tetrazolium salt, 2-(2-methoxy-4-nitrophenyl)-3-(4-nitrophenyl)-5-(2,4-disulfophenyl)-2H-tetrazolium, monosodium salt (WST-8) to a yellow formazan dye by dehydrogenase reactions. The amount of formazan dye generated will be equivalent to the number of living cells [106]. The study by Li et al. [107] reported the antiproliferative activity of iakyricidins A–D (produced by *Streptomyces iakyrus* SCSIO NS104) using the CCK-8 assay. In the study, the most notable compound was iakyricidin A which generated the strongest antiproliferative activity against the human renal carcinoma ACHN cells with IC_50_ of 0.02 µM, with weak antiproliferative activity against human renal carcinoma 786-O and OS-RC-2 cell lines. However, iakyricidins B–D showed weak to moderate antiproliferative activity against ACHN, 786-O and OS-RC-2 cells. In addition, Yu et al. [108] investigated the antiproliferative and cytotoxic activities of compounds isolated from *Streptomyces xiamenensis* 318 by using both the CCK-8 assay and lactate dehydrogenase (LDH) assay. The lactate dehydrogenase (LDH) assay is another common method used to determine cytotoxicity by measuring the LDH cytoplasmic enzyme released by damaged cells once the plasma membrane is destructed. This situation occurs when the cell is undergoing a process of cellular damage such as apoptosis and necrosis. The quantification of LDH activity can be done in the conversion process of lactate to pyruvate. This conversion is accompanied with the production of NADH that reduces yellow-coloured tetrazolium salt (INT) into a red-coloured water-soluble formazan-class dye can then be easily quantified. The quantity of this formazan dye directly indicates the content of LDH which is an indicator for cell damage/death [109]. In that study, the compounds, namely ikarugamycin (IC_50_ = 1.30 μM), capsimycin (IC_50_ = 3.33 μM), and capsimycin B (IC_50_ = 3.37 μM) possessed strong antiproliferative activity against pancreatic cancer cells without significantly affecting normal human pancreatic ductal cells [108].

**Figure 1:**
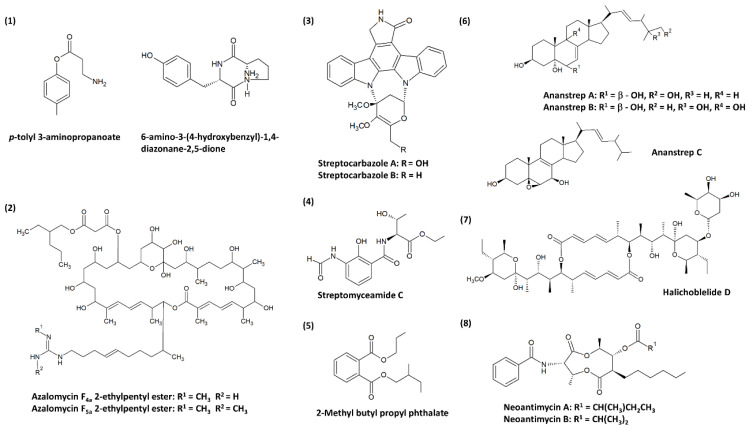
Chemical structures of the novel compounds produced by mangrove-derived streptomycetes. Chemical structures were obtained from the studies conducted by: (**1**) Xie et al. [99], (**2**) Yuan et al. [100], (**3**) Fu et al. [101], (**4**) Fu et al. [124], (**5**) Mangamuri et al. [125], (**6**) Zhang et al. [123], (**7**) Han et al. [102], and (**8**) Hu et al. [119].

The research on anticancer compounds commonly involves plants as one of the major sources. Plant-derived anticancer compounds such as vincristine of vinca alkaloid family (from *Catharanthus roseus*) and etoposide (from *Podophyllum peltatum*) are successful chemotherapy medicines [21,110]. Nevertheless, the expansion in microbial natural products research has led to the revelation of clinically important compounds such as romidepsin (from *Chromobacterium violaceum*) [111], wortmannin (from *Talaromyces wortmannin* and *Penicillium funiculosum*) [112,113], marinomycins (*Marinispora* spp.) [114], saliniquinones (from *Salinispora arenicola*) [115] and many more, which exerted potent cytotoxic capabilities. Thus, the current review attempts to provide a fresh perspective on anticancer compounds from mangrove-derived streptomycetes. There is evidence that several new compounds were successfully recovered from streptomycetes as shown in Figure 1, and this may indicate that the acquisition of streptomycetes from underexplored sources such as mangrove can be an effective strategy to search for new anticancer agents as candidates for drug development [66]. Previous findings have insinuated that mangrove-derived actinomycetes are producers of bioactive compounds of alkaloids, dilactones, macrolides, xiamycins, salinosporamides, indole alkaloids, 2-pyranones, sesquiterpenes, and phenazines [77,88]. According to the studies compiled in this review, the recovered cytotoxic compounds belonging to dilactones (e.g., antimycins), indole alkaloids (e.g., streptocarbazoles), and macrolides (e.g., azalomycins, elaiophylins) are produced by mangrove-derived streptomycetes, in which the compound families identified are consistent with the earlier findings. Antimycins can be produced by streptomycetes of various origin. For instance, *Streptomyces* sp. AZ-AR-262 [116] from a terrestrial Egyptian soil sample, *Streptomyces* sp. K01-0031 [117] from a mountain soil sample and *Streptomyces lusitanus* [118] of marine source are capable of antimycins production. However, these antimycins were initially known for their antimicrobial activities. The anticancer property of antimycins was later investigated by Hu et al. [119] on lung cancer, breast cancer, and glioblastoma cells, which subsequently demonstrated a promising outcome. Hence, this had established the potential of antimycins as lead compounds for chemotherapeutic medicines. One of the most compelling discoveries was the isolation of ergosterols from mangrove-derived streptomycetes. Ergosterols are conventionally found in fungi, sponges, and corals [120,121,122], until Zhang et al. [123] first described the isolation of cytotoxic ergosterols produced by a *Streptomyces* strain. This is likely owed to the presence of cholesterol oxidase in streptomycetes which is important for the transformation of sterols. Overall, most of these compounds in Table 1 have unique structures and possible medicinal use, therefore this scenario further accentuates the status of mangrove-derived streptomycetes as astounding natural product producers.

## 5. Crude Extracts of Mangrove-Derived *Streptomyces* spp. with Anticancer/Cytotoxic Activity

As most research focuses on the investigation of bioactivities of pure compounds extracted from streptomycetes, it is also important to acknowledge other studies that performed bioactivity screening on crude extracts of streptomycetes (Table 1). Among these different crude extracts, it can be observed that the crude ethyl acetate extract of *Streptomyces* sp. ACT01 isolated from the Indian mangrove ecosystem demonstrated potent cytotoxicity against MCF-7 and MDA-MB-231 breast cancer cell lines with low IC_50_ values of 19.49 µg/mL and 32.79 µg/mL, respectively [126]. Similarly, Tan et al. [127] also revealed that the ethyl acetate fraction of *Streptomyces* sp. MUM256 isolated from a Malaysia mangrove forest was the most cytotoxic against HCT-116 colon cancer cells (IC_50_ value of 88.44 μg/mL). It was also reported that this particular fraction of *Streptomyces* sp. MUM256 crude extract was capable of: (a) reducing the proliferation of HCT-116 cells by causing cell-cycle arrest in the G1 and G2 phases, and (b) inducing mitochondrial-mediated apoptosis in HCT-116 cells.

Additionally, Ser et al. [30] reported the cytotoxic activity showed by the methanolic extract of a novel strain, *Streptomyces malaysiense*, isolated from a mangrove forest in Malaysia. The extract was active against four types of human cancer cell lines: colon cancer cell lines HCT-116 and HT-29, cervical cancer cell line Ca Ski, and lung cancer cell line A549. The extract exhibited the greatest cytotoxicity against HCT-116 with a cell viability of 48.8% at the highest tested concentration of 400 µg/mL. Notably, the diversity and bioactivities of streptomycetes from mangrove environments in Malaysia were investigated by Law et al. [75]. High-throughput in vitro cytotoxic activity screening was conducted in the study and the results showed that 11 *Streptomyces* isolates exhibited significant cytotoxicity against several human colon cancer cell lines such as SW480, HT-29, HCT-116, and Caco-2.

In most situations, crude extracts’ cytotoxic activity is often considered a preliminary outcome that may not reflect the true activity. The promising cytotoxicity exhibited by these extracts should not be neglected either. It could be worthwhile to perform additional experiments on these extracts to further evaluate the compounds present responsible for the anticancer-related property. For some cases, the crude extract’s chemical profiling is performed with the application of gas chromatography–mass spectrometry (GC–MS) before conducting any purification of the compound. Such an approach can be observed in several studies that reported the GC–MS detection of various phenolic and pyrrolopyrazine compounds in *Streptomyces* strains such as *Streptomyces pluripotens*, *Streptomyces malaysiense*, *Streptomyces colonosanans*, *Streptomyces* sp. MUM256, and MUM265 isolated from Malaysian mangroves [29,30,33,84]. Phenolic compounds and pyrrolopyrazines (cyclic dipeptides) are regarded as compounds capable of exerting anticancer activity and other biological activities such as antioxidant and antimicrobial [128,129,130,131]. 

**Table 1:** Summary of studies pertaining to the anticancer/cytotoxic activity of mangrove-derived *Streptomyces* during the period 2008–2019.

No.	Author	Strain	Source	Compound/Crude Extract	IC_50_ (µg/mL)
**Pure Compound**					
1.	Xie et al. [99]	*Streptomyces* sp. 124092	Mangrove rhizosphere soil of *Heritiera littoralis* (China)	(1) *p*-tolyl 3-aminopropanoate * (2) 6-amino-3-(4-hydroxybenzyl)-1,4-diazonane-2,5-dione * (3) *n*-butyl mannoside (4) daidzein	SMMC-7721 cells: (1) 22.0; (2) 60.0; (3) 13.6; (4) 6.0
2.	Yuan et al. [100]	*Streptomyces* sp. 211726	Mangrove rhizosphere soil of *Heritiera globosa* (China)	(1) Azalomycin F_4a_ 2-ethylpentyl ester * (2) Azalomycin F_5a_ 2-ethylpentyl ester *	HCT-116 cells: (1) 5.64; (2) 2.58
3.	Fu et al. [101]	*Streptomyces* sp. FMA	Mangrove soil (China)	(1) Streptocarbazole A * (2) Streptocarbazole B *	HL-60 cells: (1) 0.67 ^a^ A549 cells: (1) 2.41 ^a^ HeLa cells: (1) 16.61 ^a^; (2) 10.47 ^a^
4.	Cibi and Nair [132]	*Streptomyces parvulus* CBJ1	Mangrove soil (India)	Actinomycin D	A549 cells: 0.52
5.	Fu et al. [124]	*Streptomyces antibioticus* strain H74-21	Sediment from a mangrove site (China)	Streptomyceamide C *	MCF-7 cells: 27.0
6.	Mangamuri et al. [125]	*Streptomyces cheonanensis* VUK-A	Sediment from Coringa mangrove ecosystem (India)	2-Methyl butyl propyl phthalate *	MCF-7 cells: 1391.7 MDA-MB-231 cells: 278.34 HeLa cells: 27.834 OAW-42 cells: 278.34
7.	Zhang et al. [123]	*Streptomyces anandii* H41-59	Sediment from a mangrove district (China)	(1) Ananstrep A * (2) Ananstrep B * (3) Ananstrep C * (4) Ergosta-7,22-diene-3β,5α,6β,25-tetraol (5) Ergosta-7,22-diene-3β,5α,6β-triol (6) Ergosta-7,22-diene-3β,5α,6α-triol (7) Ergosta-7,22-diene-3β,5α,6β,9α-tetraol (8) 5α,6α-epoxy-ergosta-8(9),22-diene-3β,7α-diol (9) 5α,6α-epoxy-ergosta-8(14),22-diene-3β,7α-dio (10) Ergosta-8(9),22-diene-3β,5α,6β,7α-tetraol (11) Ergosta-8(14),22-diene-3β,5α,6β,7α-tetraol (12) Ergosta-5,7,22–triene-3β-ol 5β,6β-epoxy-ergosta-8(14),22-diene-3β,7β-diol	MCF-7 cells: all compounds exhibited > 50; except (3) 18.1, (8) 24.3, (10) 17.3, and (11) 27.4 SF-268 cells: all compounds exhibited > 50; except (3) 13.0, (8) 15.5, (10) 27.8, and (11) 25.1 NCl-H460 cells: all compounds exhibited > 50; except (3) 23.5, (8) 19.8, (10) 23.7, and (11) 23.7
8.	Han et al. [102]	*Streptomyces* sp. 219807	Mangrove soil (China)	(1) Halichoblelide D * (2) 2-methyl-11,11′-*O*-dimethylelaiophylin (3) 2-methylelaiophylin (4) Elaiophylin (5) 11-*O*-methyl-elaiophylin (6) 11,11′-*O*-dimethylelaiophylin (7) Efomycin G	HeLa cells: (1) 0.27 ^a^; (2) 1.20 ^a^; (3) 0.30 ^a^; (4) 0.30 ^a^; (5) 0.59 ^a^; (6) 0.60 ^a^; (7) 0.60 ^a^ MCF-7 cells: (1) 0.30 ^a^; (2) 2.26 ^a^; (3) 0.30 ^a^; (4) 0.19 ^a^; (5) 1.00 ^a^; (6) 1.01 ^a^; (7) 0.80 ^a^
9.	Hu et al. [119]	*Streptomyces antibioticus* H12-15	Sediment from a mangrove district (China)	(1) Neoantimycin A * (2) Neoantimycin B * (3) Antimycin A_1ab_ (4) Antimycin A_2a_ (5) Antimycin A_9_	MCF-7 cells: (1) >50; (2) >50; (3) 18.1; (4) 36.4; (5) 26.1 SF-268 cells: (1) 33.6; (2) 41.6; (3) <1.6; (4) <1.6; (5) <1.6 NCl-H460 cells: (1) >50; (2) >50; (3) 21.7; (4) 43.7; (5) 15.5
**Crude Extract**					
10.	Ravikumar et al. [126]	*Streptomyces* sp. ACT01, ACT02, ACT03, ACT04, and ACT 05	Mangrove sediment from Manakkudi mangrove ecosystem (India)	Crude ethyl acetate extract	MCF-7 cells: (ACT01) 19.49; (ACT02) 29.94; (ACT03) 75.54; (ACT04) 66.04; (ACT05) 92.64 MDA-MB-231 cells: (ACT01) 32.79; (ACT02) 69.84; (ACT03) 84.09; (ACT04) >100; (ACT05) >100
11.	Ser et al. [33]	*Streptomyces pluripotens* MUSC 137^T^	Mangrove soil (Malaysia)	Crude methanol extract	MCF-7 cells: 61.33 HCT-116 cells: 83.72 A549 cells: 147.20 Ca Ski cells: 300.50 HT-29 cells: 300.98 Caco-2, SW480 and DU145 cells: >400
12.	Tan et al. [29]	*Streptomyces* MUM256	Mangrove soil (Malaysia)	Crude methanol extract	HCT-116 cells: 292.33 HT-29, Caco-2, SW480, MCF-7, A549, DU 145, and Ca Ski cells: ^b^ N.P
13.	Sanjivkumar et al. [133]	*Streptomyces olivaceus* (MSU3)	Mangrove rhizosphere soil of *Rhizophora mucronata* (India)	Crude ethyl acetate extract	MCF-7 cells: 88.26 HT-29 cells: 104.81
14.	Ser et al. [30]	*Streptomyces malaysiense* MUSC 136^T^	Mangrove soil (Malaysia)	Crude methanol extract	HCT-116, HT-29, A549, and Ca Ski cells: ^b^ N.P.
15.	Law et al. [84]	*Streptomyces colonosanans* MUSC 93J^T^	Mangrove soil (Malaysia)	Crude methanol extract	HCT-116, HT-29, Caco-2, and SW480 cells: ^b^ N.P.
16.	Ser et al. [134]	*Streptomyces gilvigriseus* MUSC 26^T^	Mangrove soil (Malaysia)	Crude methanol extract	HCT-116, HT-29, Caco-2, and SW480 cells: ^b^ N.P.
17.	Law et al. [135]	*Streptomyces monashensis* MUSC 1J^T^	Mangrove soil (Malaysia)	Crude methanol extract	HCT-116 and SW480 cells: ^b^ N.P.
17	Law et al. [75]	11 strains of *Streptomyces* sp.	Mangrove soil (Malaysia)	Crude methanol extract	HCT-116, HT-29, Caco-2, and SW480 cells: ^b^ N.P.
18	Tan et al. [127]	*Streptomyces* sp. MUM256	Mangrove soil (Malaysia)	Ethyl acetate extract (further study from Tan et al. [29])	HCT-116: 88.44
19	Tan et al. [3]	*Streptomyces* sp. MUM 265	Mangrove soil (Malaysia)	Crude methanol extract	HT29 and Caco-2: ^b^ N.P.

* new compound identified in the study. ^a^ IC_50_ value is converted to µg/mL based on the calculation of given data in the study; ^b^ N.P = IC_50_ value is not provided in the study. Note: cytotoxic activity was analysed using the MTT method involving human cancer cell lines (SMMC-7721 = hepatocarcinoma cell line; A549 and NCl-H460 = lung cancer cell lines; HCT-116, HT-29, and SW480 = colon cancer cell lines; HL-60 = leukaemia cell line; MCF-7 and MDA-MB-231 = breast cancer cell lines; SF-268 = glioblastoma cell line; Ca Ski = cervical cancer cell line; DU 145 = prostate cancer cell line; HeLa = cervical cancer cell line; OAW-42 = ovarian cancer cell line).

## 6. Limitations and Suggestions for Future Research

The research on the anticancer property of mangrove-derived streptomycetes is still in the preliminary stage. Moreover, all relevant studies compiled in Table 1 were conducted on mangrove areas in Asian countries, mainly China, Malaysia, and India. The present information is restricted to these countries, and it is encouraged to explore the anticancer potentials of streptomycetes isolated from mangroves of countries in other regions such as South America, Africa, Australia, and North America [136]. It is also necessary to carry out the in-depth research and exploration of the pure compounds or crude extracts with anticancer activity to provide a better understanding of their potential as chemotherapeutic drugs. For pure compounds, additional in vitro and in vivo analyses are required to investigate the compound’s underlying anticancer mechanisms on the cancer cells (e.g., apoptotic pathway) [77].

Collaborative approaches for interdisciplinary research through innovative techniques for more extensive investigation on both novel strains and bioactive compounds are of significance for discovering new therapeutic agents to improve human health. Bioassay-guided fractionation and purification can be applied to identify the possible pure compound(s) from crude extracts responsible for bioactivity of interest [137]. A crucial step in this process is the separation of extracts into several fractions. This can be done by several chromatography techniques such as high-performance liquid chromatography (HPLC), thin-layer chromatography (TLC), and gel chromatography. The eluted fractions will be tested to search for the bioactivity of interest accordingly. Occasionally, the fraction that is determined to be the most bioactive component of the extract can be further fractionated until the pure active compound is isolated for identification. Finally, the detection and elucidation of the structure of the active compound can be done using a combination of these techniques—mass spectrometry, nuclear magnetic resonance (NMR), or infrared spectroscopy (IR) [138].

It has always been a challenge to cultivate all naturally occurring microbes due to the presence of viable but non-culturable states of bacteria; over 99% of them resist being cultivated in the laboratory. Thus, their chemical richness remain untapped [20]. This could greatly limit the isolation and discovery of potentially useful bacteria. Nonetheless, research involves utilization of recent molecular techniques could enable the detection and identification of non-culturable strains in environmental samples via extraction and sequencing of nucleic acids (ribosomal rRNA or DNA) [20,76,139,140]. An important technique known as “metagenomics” is commonly used for discovering uncultivable microorganisms from different environments. In this technique, microbial DNA will be isolated from a selected environmental sample, and the DNA sequences obtained will be the representation of microorganisms present in the sample, which may contain hundreds or thousands of microbial species per metagenome [141,142]. By taking the study conducted by Kaur et al. [143] as an example, 16S metagenomic approach was applied to investigate the microbial diversity of a Manikaran (India) hot spring. A culture-independent method will be more reliable for the study of bacteria from the extreme environment as their recovery using the traditional culture method may be compromised due to the unique growth conditions adapted by these bacteria in hot springs (e.g., a high temperature of 30–96 °C, nutrients level, etc.). This metagenomic study had successfully revealed the diversity of bacteria in the Manikaran hot spring which comprised those belonging to the phyla *Actinobacteria*, *Acidobacteria*, *Bacteriodetes*, *Cyanaobacteria*, *Chloroflexi*, *Chlorobi*, *Deinococcus*, *Proteobacteria*, *Rhodothermeota*, and *Verrucomicrobia*.

Each type of environmental sample offers a collection of DNA sequences that may encompass gene clusters responsible for natural products’ biosynthesis [144,145]. Genomic studies of streptomycetes could then provide a genetic basis to enhance the understanding of the secondary metabolism and the production of target bioactive metabolites, thus creating an opportunity to discover novel bioactive compounds. The advent of next-generation sequencing (NGS) technology allows the high throughput sequencing of bacteria’s whole genome with improving read lengths. This genomic information can be utilized for biosynthetic gene clusters mining using sophisticated computational software, e.g., antibiotics and Secondary Metabolite Analysis Shell (antiSMASH), to accelerate the annotation of metabolite gene clusters capable of natural product biosynthesis, hence allowing the study of natural product biosynthetic pathway [146,147]. Interestingly, the whole genome sequence of *Streptomyces monashensis* MUSC 1J^T^ (Table 1) was examined by Ser et al. [148] using antiSMASH analysis and the data showed that there was 59 biosynthetic gene clusters affiliated to the biosynthesis of secondary metabolites. The analysis had detected a gene cluster related to the biosynthesis of desferrioxamine B (83% similarities) in the genome of *Streptomyces monashensis* MUSC 1J^T^. Desferrioxamine was used to treat iron overload and has been associated with anticancer properties such as diminishing leukaemia growth and neuroblastoma [149]. The presence of this gene cluster reemphasizes the bioactive value of *Streptomyces monashensis* MUSC 1J^T^ which possessed antioxidant activity and cytotoxic properties against human colon cancer cells [135,148,149]. Likewise, the whole genome of *Streptomyces gilvigriseus* MUSC 26^T^ was sequenced and analysed by Ser et al. [67] and thereby disclosed the presence of a biosynthetic gene cluster associated with the production of desferrioxamine B (40% similarities) via antiSMASH, whilst Prediction Informatics for Secondary Metabolomes (PRISM) and Bacteriocin Genome Mining Tool (BAGEL3) detected several biosynthetic gene clusters that related to class I lantipeptide, lasso peptide, and bacteriocin production.

Subsequently, the information obtained from genomic analysis could improve the production of the target compound [150]. This can be achieved by several methods, for example, the overexpression of structural or regulatory genes involved in the biosynthesis of target metabolites, and the activation and expression of silent biosynthetic gene clusters in homologous or heterologous host. A study conducted by Malla et al. [151] reported that the overexpression of regulatory genes *dnrl*, *dnrN*, and *afsR* combination resulted in a 4.3-fold increased production of doxorubicin by *Streptomyces peucetius*. Typically, the biosynthetic gene clusters involved in the biosynthesis of the metabolite of interest can be identified using various bioinformatics tools. Then, the activation of the target biosynthetic gene clusters can be done through two main strategies [152,153]: (1) homologous expression, which involves eliciting the gene expression in the native host through the manipulation of endogenous transcriptional, translational or metabolic elements by mutation or (bio)chemical stimulants; (2) heterologous expression, which involves the activation of biosynthetic gene clusters in a non-producing ‘clean’ host through direct cloning or gene synthesis. Once the targeted compound is produced, the process is followed by the identification, isolation, and structural elucidation of the corresponding compound [152,154]. The study of biosynthetic pathways can provide a better understanding regarding the mechanisms of production of chemo-preventive lead compound(s) by streptomycetes.

## 7. Conclusions

Natural products play significant roles in improving human health and wellbeing, including their application in cancer prevention and treatment regimens. The plethora of bioactive secondary metabolites produced by streptomycetes has shown great biopharmaceutical potential. Mangrove environments posed as underexplored habitats have been recently exposed as rich sources of bioactive streptomycetes. The current review shows that mangrove-derived streptomycetes are promising producers of compounds with anticancer properties, some of which are new compounds such as streptocarbazoles A and B, streptomyceamide C, neoantimycins A, and B. In addition to that, extracts of these mangrove-derived streptomycetes have demonstrated great anticancer potential. Thus, this urges the need to expand the research in this area, which involves several approaches such as the purification and identification of target compounds as well as the genomic studies and analysis of biosynthetic gene clusters of the active strains. There is limited knowledge about the anticancer activity mechanisms of these compounds in cells and living organisms; therefore, it is also important to conduct further investigations to determine the effectiveness of the compounds as potential new drugs or lead compounds for cancer treatment.

**Authors Contributions:** The literature search, data extraction, and manuscript writing were performed by J.W.-F.L., L.N.-S.L., V.L., and L.T.-H.T., while S.H.W., K.-G.C., N.-S.A.M., and L.-H.L. provided vital guidance, insight, and technical support for the completion of the project. L.-H.L. and J.W.-F.L. founded the research project. All authors have read and agreed to the published version of the manuscript.
